# Acceptability of AAI from the Perspective of Elderly Clients, Family Members, and Staff—A Pilot Study

**DOI:** 10.3390/ijerph17165978

**Published:** 2020-08-18

**Authors:** Kristýna Machová, Radka Procházková, Petra Konigová, Ivona Svobodová, Lucie Přibylová, Mariana Vadroňová

**Affiliations:** 1 Department of Ethology and Companion Animal Science, Faculty of Agrobiology, Food and Natural Resources, Czech University of Life Sciences, 165 00 Prague, Czech Republic; knig.f@seznam.cz (P.K.); svobodovai@af.czu.cz (I.S.); pribylova@af.czu.cz (L.P.); maryvadronova@seznam.cz (M.V.); 2 Department of Statistics, Faculty of Economics and Management, Czech University of Life Sciences, 165 00 Prague, Czech Republic; prochazkova@pef.czu.cz

**Keywords:** animal-assisted interventions, client’s health, dog

## Abstract

Although animal-assisted interventions (AAIs) are increasingly part of comprehensive rehabilitation and many of its effects are already well described, the methodology for performing AAI depends on the specific patient, animal, and treatment objective. Acceptability of AAI from all involved members is a little explored area. Thus, 214 respondents (32 AAI clients, 146 family members, and 36 healthcare and social care workers; 98 males, 116 females; mean age 46.3 years (±16.5 SD)) completed a list of statements focused on AAI with a dog. This list was distributed directly in nursing homes, retirement homes, and in households with home hospice care. All statements were rated on a Likert scale of 0–3. The results show that AAI is generally very well received, with over 90% of respondents considering AAI to be beneficial. The perception of AAI and trusting the handler with their dog was evaluated very positively, as well as possible concerns about hygiene. The results were in many cases affected by demographic factors of the respondents (age, gender, role in AAI, education, and size of settlement). It seems appropriate in future studies to focus on the attitude of individual groups, and thus advance the methodology of implementing AAI.

## 1. Introduction

Animal-assisted interventions (AAI) are increasingly used in the field of health care, education, and social care. Specially trained animals are included in these interventions, which are adapted to the specific needs of the client [1]. Depending on whether the activity aims to distract or please clients, these interventions are called animal-assisted activities (AAA) or, in case of goal-oriented, planned, and structured therapeutic intervention directed and/or delivered by health, education, or human service professionals, it is called animal-assisted therapy (AAT). The progress of these interventions is measured and recorded in professional documentation [2]. AAI is a technique that can enhance the course of treatment and increase the overall effectiveness of the rehabilitation process. The animal plays the role of the so-called cotherapist [3].

In the field of health and social care, the most commonly used animals are dogs because they can visit clients directly in hospitals, daycare centers, or homes for the elderly [4]. The presence of a dog in these facilities offers many benefits, e.g., for clients who stay there for a longer period or who suffer from mental health problems [5,6].

AAI can improve client’s specific functions in the physical, social, emotional, or cognitive areas [7]. Some studies have shown the effect of AAI on reducing the physiological manifestations of pain, slowing heart rate and breathing, lowering body temperature and blood pressure, and reducing the overall stress response [8]. In the treatment of mental illness and cognitive deficits, AAI can help clients maintain attention, increase their self-confidence, reduce anxiety and loneliness, or even improve verbal and nonverbal communication [9]. Several studies have reported the benefits of AAI in individual or group therapy [10]. Conducting AAI can also affect the mood of people in the whole hospital department, as well as communication between the client and staff or other patients residing in the hospital department [11,12]. When following the recommended instructions, no negative effects on the hygienic or epidemiological issues in the department where the AAI took place was observed [13].

In all interventions, the therapeutic team consists of a therapy dog, volunteers, or professional externs, who are working under the supervision of facility staff. The therapy dog may also belong to somebody of the staff [7]. The responsible persons have to carefully select a healthy animal, which is ideally certified and has documents proving the state of its health. However, equally important is the choice of a suitable client, who must be informed about the presence of the dog in advance and agree with it. The dog handler and staff need to be also informed in advance about the client’s possible problems, concerns, allergies, or phobias. In that case, it is not suitable for the clients to take part in the intervention program with the participation of an animal [10]. All these conditions and rules are based on a resolution of the International Association of Human-Animal Interaction Organizations (IAHAIO). Moreover, the handler of a therapy dog, who passes with their dog an IAHAIO exam, is obliged to respect the rules. It is possible, as per regional regulations, to pass the tests at an organization operating in the given state, and the granting of the animal’s access to the facility always depends on the willingness and helpfulness of the given subject [14].

It seems that the therapeutic goals and approaches in AAI are starting to be well-formed and AAI legislation is already well defined, as well as accepted by facilities. Many publications have confirmed the positive impact of AAI on clients [15], although some studies have raised doubts due to unclear or inconsistent methodology [16]. Furthermore, there is, despite AAT’s benefits and complexity, a lack of knowledge about its objectives and therapeutic applications, as health professionals and patients’ relatives seem to perceive it only as a distraction or entertainment [17].

The clients’ opinion on AAI, as well as of their family members or staff, is still a not thoroughly explored area. Some studies have looked at the staff’s views on AAT, e.g., in military facilities [18]. Other studies evaluated not just the effect of AAI and rehabilitating a particular problem but also the satisfaction with AAI from the perspective of the staff [19,20], parents [20], or children who participated in the AAI sessions [21]. Hinic et al. [19] reported great satisfaction from parents whose children participated in AAI and, furthermore, the parents’ desire to repeat the interventions. The same study also reported that parents found the therapy beneficial not only for their children but also for themselves, as they have been able to relax more. Furthermore, parents observed increasing children’s self-esteem correlated with regular dog therapy [20].

This study aimed to investigate the attitude of clients, healthcare professionals, and family members towards AAI in health and social care facilities, and evaluate whether they perceive these interventions positively or negatively. Next goal of the study was to assess whether the answers obtained are influenced by a factor such as age, education, size of the settlement, gender, or role in relation to AAI.

## 2. Materials and Methods

### 2.1. Participants

In total of 214 respondents participated in the study. Out of 214 respondents, 98 (46%) were men and 116 (54%) were women. Of these respondents, 32 (15%) were AAI clients (20 males and 12 females), 146 (68%) were family members (74 males and 72 females), and 36 (17%) were healthcare and social care workers (3 males and 33 females). According to the sociodemographic data, 32 respondents (15%) lived in a village with less than 500 inhabitants; 162 respondents (76%) resided in a small town with 500–10,000 inhabitants, and 20 respondents (9%) were from a larger city with more than 10,000 inhabitants. The size of settlements was differentiated according to the categories of settlement structure in the Czech Republic. From the 214 participants of this study, 149 (70%) completed high school education, 35 respondents (17%) were university graduates, and 28 respondents (13%) received postgraduate education.

The mean age of the entire sample of respondents was 46.3 years (+/− 16.5 SD). The study involved 44 respondents in the age category up to 30 years, 31 respondents in the category 30–40 years, 56 respondents in the category 40–50 years, 41 respondents in the category 50–60 years, 21 respondents in the range 60–70 years, and 21 respondents were more than 70 years old. The clients’ average age was 60.3 (+/− 16.9 SD), family members was 40.4 years (+/− 11.5 SD), and health and social workers was 44.6 years (+/− 15.9 SD). The mean age of the clients was statistically significantly different from the mean age of the family members and staff (*p* < 0.001). The men’s average age (47.7) did not statistically differ from women’s mean age (45.0) as the *p*-value was very high (*p* = 0.2401), moreover, no statistically significant difference was found according to its variability (*p* = 0.3487). 

All participants were contacted directly in the social and health care facility and consented to the use of the obtained data. All participants have been instructed on how to complete the list of statements and were able to take as long as they needed to complete it. In the case of older respondents, it was ensured that they had visual aid if they needed to. All respondents completed the list of statements individually and without any assistance. Respondents with cognitive deficits did not participate in the study. All respondents had experience with AAI in various roles as active participants, employees, or family members of clients. The study was approved by the CULS Ethics Committee, and all respondents signed an informed consent. The facilities in which the study took place also agreed to participate in the study and allowed their employees to communicate their opinion on AAI.

### 2.2. General Procedures and Measures

All data were collected from March to May 2019 in the north of Czech Republic (Ústí nad Labem and neighborhood). The list of statements (42 statements) was distributed in the printed form directly in a nursing home, in a retirement home, and in households where home hospice care takes place. Respondents were recruited in these places. Therefore everywhere, where AAI with therapy dogs takes place. Therapy dogs visit those facilities at different frequencies and intervals. The average number of AAI visits was 15 per client, with the average length of 30 min. AAI sessions were performed with different therapy dogs and professional handlers, but each client met the same dog several times in the path. All therapy dogs and their handlers passed the necessary tests, these teams (dog and its handler) passed the needed exam together, and are certified to perform AAI, which should be a guarantee of professional implementation of AAI in comparable quality.

The distribution of the list of statements lasted for 2 weeks with the consent of the facilities. The average time needed to complete the list of statements was 15 min. Each respondent completed a record sheet in which they became acquainted with the study and signed informed consent. The sheet contained demographic questions about gender, age, schooling years, size of the settlement, and their role in this study. The next part of the paper was focused on the subjective evaluation of 45 statements. All statements were rated in the same way on a Likert scale of 0–3, where 0 meant the respondent was unable to assess the statement, 1 meant “completely disagree” with the statement, 2 meant “slightly agree with the statement,” and 3 meant “completely agree with the statement.” The statements focused on whether the respondents perceived AAI positively or not, subsequently, individual statements focused on the possible effects of AAI, such as client pleasure, alleviation of loneliness, simplification of contact with the environment, building relationships with employees, and more. Respondents were also asked whether AAI in the facility did raise any concerns, either from clients, family members, or employees. If the respondent already had any experience with AAI, then the statements focused on its evaluation, frequency, and preferences of the form of AAI (group/individual).

### 2.3. Data Analysis

The obtained data matrix contained general information about the respondents (gender, age, education, role, and size of permanent settlement) and the degree of their agreement or disagreement (scale 0–3) with 45 statements related to AAI with a dog. Before the actual statistical analysis, a survey analysis of the data was performed to verify the assumptions for subsequent statistical processing. Only the quantitative variable (age) was assessed and tested across categories (gender, education, size of place of residence, and role) using a two-sample *t*-test and one-way analysis of variance. 

The results of the list of statements were evaluated using absolute and relative frequencies of responses. Furthermore, the influence of selected factors (role, gender, age category, education, and size of permanent settlement) on the respondents’ attitudes towards AAI was assessed using the analysis of association and contingency tables. Statistically significant dependence of attitudes on selected factors was tested using Pearson’s χ^2^ test of independence. Statistical significance was set at *p* < 0.05. In the case of a proven statistically significant dependence, the strength of this dependence was measured and assessed using Pearson’s contingency coefficient or Cramer’s contingency coefficient (for cases where one variable had more than 2 categories) and association coefficient and coefficient φ—Phi coefficient (for cases where each variable had exactly 2 categories).

Graphs of interactions were used to visualize the results of the tests of qualitative features. STATISTICA 13.2 software (StatSoft, version Cz. 7, Tulsa, OK, USA,) for Windows was used for statistical analysis.

## 3. Results

### 3.1. Factors Significantly Influencing Opinions and Attitudes towards AAT

Several factors of significant influence have been found. Summary results of significance of individual factors against individual statements can be found in Table 1. A *p*-value < 0.05 was reported when a statistically significant dependence was observed. Empty spaces are present when no test was possible for various reasons, mostly for small sample sizes. All these factors influenced the obtained responses with mild or medium strength. Table 2, Table 3, Table 4, Table 5, and Table 6 contain an overview of the specific percentage of responses for statements for which the given factor proved to be significant, as well as the percentage distribution of respondents’ responses for these statements. The following text describes some important factors and issues which the factor affected. These statements are always marked with the letter S and the number of the statement in Table 1.

Age is a factor influencing the results of the statement of whether the therapy dog can cause allergies (S4), which could complicate the functioning of the hospital department (*p* = 0.012; V = 0.230). For example, from the category of respondents under 30 years, 31.82% are not afraid of allergies, 29.55% cannot judge it, and 38.64% acknowledge this possibility. On the other hand, 28.57% of respondents aged 60–70 cannot assess this fact, and the remaining 71.43% slightly agree with the possibility of the dog causing allergies during AAI. Age also affected the response to the statements regarding the dog being a significant motivating element during therapy (S33) (*p* < 0.001; V = 0.293). None of the respondents disagree with this statement, 40.91% of respondents under the age of 30 completely agree with this statement, and nearly 30% slightly agree with this statement. In the category of 30–40 years, 70.97% completely agree and 12.90% slightly agree. In the category of 50–60 years, 53.76% completely agree with the statement, 17.07% express a slight agreement, and 29.00% cannot assess the statement.

Furthermore, age determined the responses regarding the friendlier and less formal environment, which was created by the presence of a dog (S34) (*p* < 0.001; V = 0.261). In the under-30-years old category, 56.82% of respondents completely agree with this statement, 67.74% in the 30–40 category, 19.05% in the 60–70% category, and 4.76% of the over-70 category. Furthermore, this statement was slightly agreed on by 22.73% of respondents under 30, almost 10.00% of respondents in the category 30–40 years, 38.10% in the category 60–70 years, and 47.62% in the category over 70 years. On the contrary, 2.27% of respondents under 30 do not agree with the statement, as well as almost 10% in the category 30–40 years, 1.79% in the category 40–50 years, 12.20% in the category 50–60 years, 4.76% in category 60–70 years, and 4.76% from the category over 70 years. The strength of these dependencies was evaluated as low. All statements where age was a statistically significant factor are indicated in Table 2.

Significant dependence regarding the residence factor was found in the matter of suitability of the dog in the psychiatric department (S23) (*p* = 0.038; V = 0.154), where no one expressed disagreement. Complete agreement was expressed by 40.63% of respondents from villages, 63.58% of respondents from small towns, 45.00% of respondents from large towns, and 5.00% of the respondents living in a big city. A large proportion of respondents stated that they could not respond to the statement (40.63% from a village, 25.93% from a small town, and 50.00% from a large town). The residence factor also influenced the respondents’ reaction to the statement mapping the acceptance of AAI by clients and their behavior towards the therapy dog (S31) (*p* = 0.001; V = 0.204). Exactly 50.00% of the village population completely agreed with this statement, as did 63.58% from a small town and 35.00% from a large town. The inability to express their opinion was stated by 34.38% of the inhabitants of a village, 25.31% of the inhabitants of a small town, and 2.00% of the inhabitants of a big town. Similar to age, the residence was also observed as a significant factor in statements focused on the dog being an ideal motivation during therapy (S33) (*p* < 0.001; V = 0.251). A considerable difference was observed between responses to this statement, where complete agreement with this statement was expressed by less than 10% of the village residents, 51.58% of the inhabitants of a small town, and 25.00% of the residents of a large town. Slight agreement was expressed by 50.00% of the population of a village, 18.52% of the population of the small town, and 25.00% of the population of the large town. No respondents declared that they did not agree with the statement. However, 40.63% of the village population could not assess this issue, as well as 29.63% of the population of a small town and half of the population of a large town. The statement whether the presence of a therapy dog creates a friendlier and more pleasant environment (S34) (*p* < 0.001; V = 0.295) was completely agreed upon by 3.13% of respondents living in a village, 57.47% of small-town residents, and 30.00% inhabitants of a large city. A slight agreement with the statement was then expressed by 46.88% of the village residents, 15.43% of the population of a small town, and 25.00% of a large town. All statements where residence was a statistically significant factor are indicated in Table 3.

Education proved to be a significant factor in 21 statements. A complete overview of these statements and a specific distribution of responses can be found in Table 1 and Table 4. For example, in the statement that the dog worries the staff and is, therefore, more stressed (S15), a slight agreement was given by 54% of respondents who completed postgraduate education and 31% of respondents who completed secondary education without a diploma. On the other hand, 72% of this group completely agreed with the statement that the therapeutic dog brings relaxation to patients (S16) and staff, with which 57% of respondents with postgraduate education also completely agreed.

On average, 51% of respondents who achieved secondary education without a diploma, 33% of respondents with a high school diploma, 46% of university students, and 71% of postgraduate respondents completely agreed with the statement that the dog is an ideal means of motivation (S33). The statement that the dog creates a friendlier and less formal atmosphere in the place where AAI takes place (S34) was completely agreed on by 62% of high school-educated respondents without a high school diploma, 30% of respondents with a high school diploma, 51% of university students, and 89% of postgraduate respondents. Overall, 74% of high school-educated respondents without a diploma, 46% of respondents with a high school diploma, 57% of university students, and 86% of postgraduate respondents completely agreed with the statement regarding the suitability of attendance of a therapy dog in the psychiatry department (S23). Furthermore, 36% of high school-educated respondents without a diploma, 19% of respondents with a diploma, 17% of university students, and 21% of postgraduate respondents completely agreed with the statement that clients are more motivated to be active in the presence of a therapy dog (S35). Approximately 50% of all respondents slightly agreed with the said statement, and a very few respondents disagreed. All statements where education was a statistically significant factor are indicated in Table 4.

The gender of the respondents only did not affect responses concerning the dog’s influence on the feeling of loneliness, the positive effect of AAI in general or on the client’s psyche, their stress, willingness to go on a walk with a therapy dog, or the preference of the dog breed for AAI. Gender also did not affect the positive or negative attitude towards AAI, compensation of the absence of one’s pet, or opinion on a possible effect on the hygiene of the facility, or potential indifference. Gender was a significant factor in all responses to remaining statements with a mild or medium strength. These statements can be found in Table 1 and Table 5 with the listing of the responses for both genders. The biggest differences in the obtained responses were in regard to, e.g., the statement that the presence of the therapy dog lightens up a monotonous hospital stay (S5), where 93.10% of females and 75.51% of males completely agreed, the remaining respondents chose the option to slightly agree. Another discrepancy was in statement (S6) concerning hygienic issues caused by the dog or worries about damaging the hospital bed or room. In this case, 33.67% of males and 7.76% of females chose not to respond. Notably, 48.98% of men and 87.07% of women did not have these concerns, and 17.35% of males and 5.17% of females had mild concerns. For the statement S20 (The therapy dog is a suitable instrument to get in touch with the surrounding), 81.90% of women and 42.86% of men completely agreed and 29.59% of men and 11.21% of females slightly agreed. The remaining respondents were unable to evaluate the statement. In statement S23 concerning the suitability of the therapy dog in the psychiatric ward, 81.90% of women and 30.61% of men expressed their complete agreement. Another 24.49% of men said that they slightly agreed with the statement. Next, 61.21% of women and 38.78% of men would actively seek the therapy dog (S25). Moreover, 17.35% of men could not express an opinion as well as 39.80% of women. Other participants said they would slightly agree, and no one answered that they did not agree. A small breed (S26) would be preferred by 81.90% of women and 51.02% of men, slightly preferred by 39.80% of men and 15.52% of women. No one completely disagreed. 79.31% of women and 34.69% of men completely agreed with the statement that clients are dog-friendly and respectful. Precisely, 66.38% of women also expressed concerns about whether participation in AAI is pleasant for the dog (S32). These concerns are shared by 34.69% of men. Interestingly, 62.93% of women and 19.39% of men consider the dog to be an ideal motivating element (S33). Another 17.24% of women and 31.63% of men slightly agree with this statement. Furthermore, 76.72% of women also believe that the presence of a dog facilitates a less formal and more pleasant atmosphere (S34), with which 11.22% of men expressed their full agreement. Another 29.59% of men and 13.79% of women slightly agreed, and 12.24% of men and no woman disagreed. In addition, 99.14% of women stated that cuddling a therapeutic dog calms them down (S37), with which 75.51% male respondents fully agreed. The rest of the male respondents slightly agreed with the statement (24.49%). Further, 12.24% of men and 1.72% of women slightly agree with the statement that respondents were bothered by the presence of a dog in the facility (S41). The remaining female respondents stated that they completely disagreed with the statement. Notably, 55.10% of men also disagreed completely, and the rest of the male respondents did not express an opinion.

The role of participants (client, staff, or family member) also influenced the obtained responses with a mild or medium strength. This effect was seen in the statement regarding the influence of AAI on the monotonic course of days in the facility (S5) (*p* = 0.003; V = 0.235). For this statement, the respondents only confirmed a complete or slight agreement. Specifically, 94.00% of clients, 79.00% of family members, and 100% of staff members completely agreed with the statement. A slight agreement was then provided by 6.00% of clients and 21.00% of family members. 

Another statement, for which responses were influenced by the role of the participant was trusting the handler to reliably control their dog (S11) (*p* = 0.001; V = 0.261). Once again, only responses confirming a slight or complete agreement were obtained. A slight agreement was reported by 44.00% of the clients and a complete agreement by 56.00% of them. For family members, 36.00% agreed slightly and 64.00% completely agreed. Finally, a slight agreement was expressed by 6.00% of caregivers, and 94.00% of them completely agreed.

Other statements, in which the effect of the role was observed, were matters related to mood stimulation (S43) (*p* = 0.033; V = 0.179). In this case, only responses confirming complete agreement or the inability to assess the statement were obtained. In clients, 75% of respondents completely agreed and 25% of respondents could not assess this statement. In family members, 82% said that activities with a therapy dog could improve clients’ mood and 18% preferred to not judge. Among caregivers, 97.00% said they completely agreed that the presence of a dog improved the mood of clients and 3% said they could not judge.

In the statement if respondents will be looking forward to the next AAI, the respondents’ role was significant (S17) (*p* = 0.003; V = 0.178), with the responses confirming a slight or complete agreement. For clients, a complete agreement in 75% of the responses was observed and slight agreement in the remaining 25%. For family members, notably, 84% expressed complete agreement, and the remaining 16% slightly agreed. For staff, 97% expressed complete agreement and the remaining 3% slightly agreed.

The specific role also affected the hygienic issues associated with the implementation of AAI (S19) (*p* < 0.001; V = 0.203). Curiously, employees have the least concern about bed and room contamination. Notably, 97% of them said they were not worried and the remaining 3% said they could not assess the issue. Out of family members, 65% of respondents were not concerned, 14% were slightly concerned, and 21% could not say. The largest percentage of respondents who were unable to assess the statement were clients (31%), furthermore, 59% of them were not concerned and 9% were slightly concerned. 

A significant difference was also observed in the statement that the therapy dog is a suitable means of establishing contact with the environment (S20) (*p* = 0.001; V = 0.242). In this case, 100% of the staff respondents stated complete agreement. However, this opinion on the said statement was supported by 53% of clients and 22% of them expressed a slight agreement. Lastly, 25% of clients were unable to assess the issue. The results of the responses provided by the family members were more or less equivalent to the responses of the clients.

A similar difference was found for the statement that clients show friendly behavior and respect towards the therapy dog (S31) (*p* = 0.002; V = 0.199), where the responses of clients and family members were similar and differed from those obtained from staff. Notably, 89% of the staff expressed complete agreement, while out of clients, it was 53%. The responses to the statement that the dog is an ideal motivating element during therapy were similar. A complete agreement was provided by 89% of staff, 38% of family respondents, and 13% of clients. Slight agreement was expressed by 3% of caregivers, 24% of family members, and 47% of clients. Moreover, 41% of clients, 38% of family members, and 8% of caregivers could not assess this issue. There is a similar disagreement in the responses to the statement that the presence of a dog creates a friendlier and less formal atmosphere in the hospital. Distinctly, 91.67% of staff, 43.84% of family members, and 9.38% of clients completely agreed with this. Slight agreement was expressed by 2.78% of staff, 19.86% of family members, and 46.88% of clients. On the other hand, 6.25% of clients and 6.85% of family members disagreed with the statement, and none of the staff disagreed. Lastly, 37.50% of clients, 29.45% of family members, and 5.56% of staff could not assess the issue. 

The responses to the statement that the dog could provide emotional support to the respondent were interesting. Out of clients, 78% completely agreed and 9% agreed slightly. On the contrary, family members and staff did not agree with this statement as immensely, as 6% of staff could feel emotional support, as in the case of family. Notably, 92% of the staff and 82% of family members slightly agree with the statement. Finally, 3% of staff, 12% of family members, and 13% of clients could not assess this issue.

### 3.2. Overall Evaluation of the Obtained Responses

#### 3.2.1. Statements Regarding the Overall View on AAI

**Figure 1:**
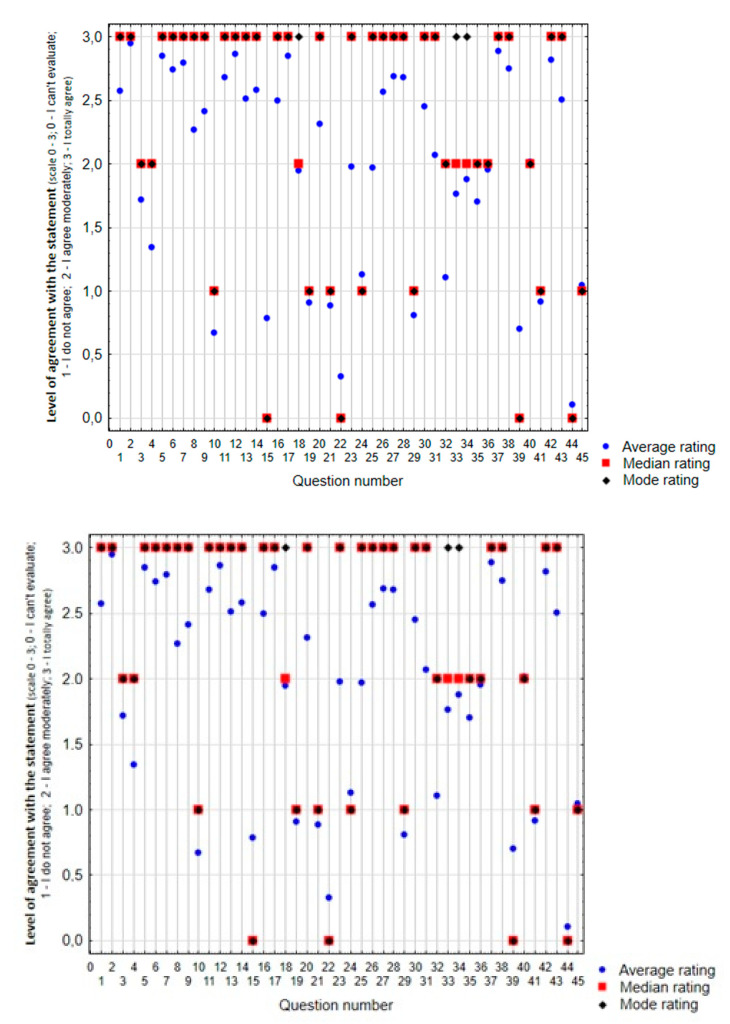
Summary of the overall results of the responses to the individual statements from the list of statements (average rating, median, and mode of evaluation on a scale of 0–3).

Regarding obtained results, 91.12% of the respondents, i.e., clients, family members, and staff, consider the presence of a dog in a social and health care facility to be beneficial, 75.23% of which find it very beneficial. Moreover, none of the respondents described it as unhelpful. The results of the responses to the individual statements (average rating, median, and mode of evaluation) are summarized in Figure 1.

All respondents (100%) are inclined to believe that a therapy dog in the social and health care facilities brings pleasure. Of these, 94.39% of respondents think that a dog in the facility brings “a lot” of pleasure. All respondents (100%) believe that the presence of a therapy dog will make the facility stay more pleasant (very beneficial for 85.10%). The vast majority of respondents (93.00%) believe that the presence of a therapy dog greatly alleviates the feeling of loneliness. A total of 83.65% of respondents reported an improvement in mood after interacting with a therapy dog, 16.36% cannot judge it. The overwhelming majority of respondents (92.50%) agreed with the statement that the presence of a therapy dog would help relax the clients and staff, furthermore, almost 65% of them strongly agreed with this statement. The vast majority of respondents (83.65%) consider a therapy dog to be a suitable means to bond with people around them.

According to the majority of respondents (67.80%), the presence of a therapy dog in the facility creates a friendlier and less formal atmosphere. On the other hand, 5.60% of respondents disagreed with this statement. Almost 27% of respondents stated they could not assess this.

Almost 90% of respondents (89.25%) reported that a therapy dog in a hospital could provide them emotional support, what is more, 16.80% of them expressed strong agreement. Furthermore, 10.70% of respondents could not assess this, as they were mostly (78.00%) family members. More than 97% of respondents said they would be happy to take the opportunity to go for a walk with a therapy dog, with 80.80% of them expressing strong agreement. None of the respondents disagreed.

The vast majority (92.50%) of respondents would agree with the possibility of participating in AAI (of which 15.40% reported very strong consent). Almost 94% of respondents agreed with the introduction of AAI programs. None of the respondents were against it, only 6.10% were not able to assess it. Notably, 73.80% of respondents are not indifferent to the presence of a therapy dog in the facility. Furthermore, 14% of respondents (only family members) expressed indifference.

All respondents (100%) said that they trust the handlers, with 68.22% opting for “very much” and 31.78% trust them moderately. The majority of respondents reported that they were not able to assess whether the quality of AAT is affected by the breed (92.06% chose I cannot judge). The vast majority of respondents (94.39%) stated that they prefer smaller breeds for AAI, with almost 68% expressing strong agreement. More than half of the respondents (51.90%) stated that they are a little worried that therapeutic work is too demanding for a therapy dog. Nevertheless, 41.12% reported that they are not able to assess this.

#### 3.2.2. Statements about Possible Concerns and Hygiene

A total of 78.50% of respondents think that a therapy dog can bring diseases and parasites to the hospital. In contrast, 14.50% have the opposite opinion. Notably, 60.75% of respondents think that a therapy dog can cause allergies and complicate the functioning of the hospital department. Only 13.08% were of the opposite opinion, furthermore, 26.17% of respondents reported they are not able to make an assessment. Nearly 70% of respondents are not at all concerned that the therapy dog might damage the bed or make it unclean, and 10.75% of respondents expressed slight concerns. Furthermore, these concerns were expressed by only 13.00% of clients, and notably, no staff reported said concerns. Only less than 5% of respondents identified a therapy dog in the hospital as inappropriate. They were mostly family members. 

Regarding the statement if respondents agree with AAI in the hospital, 73.36% of respondents strongly agreed with it. Almost 20% of respondents said they might be uncomfortable with AAI, with, again, these respondents being mostly family members (83.00%). Only less than 5% of respondents (4.66%) oppose the implementation of AAI in facilities.

## 4. Discussion

### 4.1. General Results

The overall result for the whole study is the fact that AAI with a dog in social facilities is very well received. Overall, 91.12% of respondents consider AAI to be beneficial, of which 75.23% find it very beneficial. None of the respondents expressed the opposite opinion. Similarly, in the study of Nahm et al. [22], few caregivers (8.6%) and even fewer patients (4.2%) worried that the therapy dog interfered with the work of the department. All respondents stated that AAI brings pleasure and improves the perception of social-health facilities. These responses were obtained from recipients of AAI, their family members, and staff. This finding is consistent with many other studies that report that AAI is well accepted [18,19,20,23]. Interestingly, 92% of respondents stated that they would like to take part in AAI with the participation of a dog if given the choice, and see it as an opportunity for relaxation, a motivational means, and a source of social contact. The fact that dogs alleviate the feeling of loneliness and have positive social and mental effects has been reported in other studies conducted directly on AAI recipients [24,25,26].

The authors also find it interesting that if the recipients had a choice, they would prefer AAI in the presence of a small breed dog. This finding contradicts the Crowley-Robinson and Blackshaw’s study [27], where the authors gave respondents a choice of small, medium, or large breeds, and the respondents preferably opted for small and medium breeds. In the current study, respondents were asked if they found a small breed more appropriate. They did not have the option to choose a medium breed. Thus, it seems that in the case of therapeutic dogs, a small or medium breed is preferred over a large breed. On the other hand, the study of Gazzano et al. (2013) [28] shows that the participants in their study preferred to interact with puppies and large dogs. It would seem that the preference depends on the given target group of AAI clients. However, even in the case of AAI with the participation of larger breeds, all respondents stated that they trust the handler to handle the dog properly and mediate pleasant and safe interactions with the animal.

Although the vast majority of respondents would like to participate in AAI and generally accept it very well, these same respondents were concerned about the impact on the hygiene of the facility in which AAI takes place. Three-quarters of the respondents fear that the dog could be a source of diseases of various origins, as well as cause allergies. On the contrary, the respondents were not concerned about the dog making the bed dirty. This result is interesting further because none of the interviewed staff and only 13% of clients were concerned about this fact. Despite the results obtained, however, it is always necessary to take into account that AAI may not always be everyone’s preference. In our study, 20% of respondents said they did not consider AAI to be appropriate in a facility setting. Some patients do not like dogs or have various health problems or allergies, so they do not want to participate in AAI [29]. The social-healthcare environment is sensitive to hygiene, and vulnerable populations must be protected before the introduction of AAI [20]. Attention should be paid to the possible transmission of zoonoses or allergies [13,30], which, in addition to the risk of dog bites, are the biggest concerns of AAI in hospitals [31].

In terms of the practical impact of AAI, clients state that they would like to experience AAI more often and, if possible, in the form of individual sessions. This may be because, in the case of AAI, it is often a very intimate situation, both in terms of the quality of the conversation with the client and, e.g., physical rehabilitation. Another possible explanation is that the client wants to fully enjoy the presence of the animal and make the most of the time provided for themselves. This is consistent with the findings of other studies [32,33], where the authors also evaluated individual AAIs as more beneficial than group AAIs. In addition, Nimer and Lundahl [33] state that, although not statistically significant, a meaningful difference in effect sizes favors the use of individual delivery of AAT compared with group delivery for emotional well-being outcomes. At the same time, authors Souter and Miller [7] reported that there is a lack of research comparing individual versus group interactions, despite the fact that their effectiveness is an important practical question for therapists who are planning animal interaction programs.

Clients also reported that the AAI provided has helped them reduce their stress, which is in line with, e.g., the studies of Barker et al. [34] or Wu et al. [35]. They also state that they felt more active, motivated, and in a better mood. This result is also in compliance with previous studies. The study by Motooka et al. [36] also confirms the effect on the autonomic nervous system associated with a pleasant experience, in their case, in the elderly. Respondents report this effect when petting and cuddling with a dog, similar to the study by Schuelke et al. [37], which states that petting a dog with which one is bonded promotes relaxation.

### 4.2. Factors Influencing Responses to AAI

A closer examination of the results revealed that when taking into account certain factors, respondents’ views and attitudes may often differ. The first factor considered and tested in this study was age. The opinions of older respondents often differ significantly from those of the younger generation, as stated in a study by Arsenault [38]. Life experiences, the setting of the environment and peers, or the choice of consumed media, can have a major impact on some attitudes [39]. If the age of the clients is taken into account, then the oldest respondents are the generation born just after the end of the Second World War. It is understandable that at that time, the relationship with animals could have been completely different from what exists in society now [40]. In the Czech Republic, clients report that during their youth, dogs were guarding their families, most of the time tied to a chain, and fed on leftovers from the kitchen. Sometimes they state that the dog does not belong indoors. Social, as well as personal history, is very important and should always be taken into account when implementing AAI [41]. However, it is not just about history, but also about certain mental changes that accompany old age [42]. It seems appropriate, especially for older clients, to explain everything thoroughly and patiently and to respect possible concerns. In a study by Lindström Nilsson et al. [43] on children, pediatric clients reported mixed feelings (positive and negative) before therapy and rated therapy as an unexpected event, while after AAI they all reported a positive experience. A patient approach, sufficient explanation, and time seem to help overcome any initial concerns. In the current study, older respondents were concerned, e.g., about the possibility of allergies or potential pollution of the space caused by the presence of a dog.

Another factor that may affect the perception of AAI is the size of the settlement where the respondent lives. The responses from inhabitants of small towns concerning AAI were quite surprising. The results of the analyzed survey show that it is the inhabitants of small towns (with 500 to 10,000 inhabitants) who are most positive about AAI. This concerned, e.g., the claim that the dog creates a more pleasant and less formal atmosphere in the facility or the claim that the dog can be a strong motivating element for clients in therapy. Another finding is that it seems that the inhabitants of large cities have much more definite opinions. This group much less often chose the option of not being able to assess the issue. Thus, they seem to be better informed about AAI than the inhabitants of smaller towns or villages and have a more specific idea of their opinion on the matter. The possible fears that may arise at the beginning of AAI may stem from ignorance. 

The level of education can also affect accepting AAI. Our research shows that there are significant differences in the answers given by people who have completed high school education without a diploma, with a diploma, university, or postgraduate degree. The study by Applebaum et al. [44] states that educational attainment does not affect the ownership of animal species other than birds. However, it seems that in the case of AAI, there could be some differences. Overall, respondents who have completed high school education without a diploma and respondents with a postgraduate background seem to be most optimistic about AAI. On the contrary, respondents with a high school diploma stated rather slight agreement or disagreement with the statements. It was also interesting that respondents with university and postgraduate education chose significantly less that they cannot assess the statement than respondents with a high school diploma and without a high school diploma.

Taking into account respondents by gender, women seem to be much more positive about AAI than men. They were less concerned about hygiene and environmental pollution and would actively seek out AAI. It can, hence, be expected that women would eventually apply for AAI themselves, while men would approach this option much more passively. It is, therefore, necessary to actively offer this option to male clients and address them directly. It is interesting, however, to find that 35.34% of respondents cannot respond to this statement when 17.35% of male respondents chose this option. The alternative that women would not be able to respond to the statement occurred only rarely. The other statement for which female respondents chose not to respond was a statement concerning the experience with AAI. Furthermore, women would prefer a rather small breed (81.90%), whereas almost half of the men respondents preferred small bread (51.02%). The likelihood that the AAI would bother the client seems more likely in men (12.24%), while 1.72% of women stated this possibility. The finding that women are more positive about AAI is consistent with a study by Pinto et al. [45].

Overall, all respondents were very positive about AAI. When evaluating the answers obtained according to the role of the respondent, there were again significant differences between the groups. The study by Moreira et al. [17] states that not all medical staff are familiar with what AAT is and do not have enough specific information, and therefore, perceive AAT as a form of entertainment rather than therapy. In our study, the staff commented on AAI most positively of all three groups. On the contrary, family members the least. However, it should be understood that most respondents of the two groups had a slight or absolute agreement. Therefore, it cannot be said that family members did not agree with something and the employees, on the contrary, completely agreed. Specifically, when rated on a scale of 0–3, the staff mostly rated 3 and family members opted for 2. The patients were somewhere in between. This can probably be explained by the fact that of the roles of clients, employees, and family members, family members are the most passive participants in this topic.

Furthermore, Pinto et al. [45] stated that 24.2% (N = 162) of medical staff have not heard of AAI. Nevertheless, positive views on AAI from a staff perspective are more abundant. In a study by Moody et al. [31], medical staff were rather skeptical before the introduction of AAI in the pediatric ward and feared that children would not be able to rest enough. Their views changed profoundly after the introduction of AAI in the ward. Nurses working in AAI wards have reported that ongoing AAI benefits their work in terms of easier collaboration and communication with patients [17,20], and some nurses have admitted that they have yet to learn to utilize the benefits provided by AAI [17].

This is also related to the findings we have obtained. Staff agreed almost 100% on claims that AAI improves clients’ mood, enhances the environment, motivates them, and improves communication within the department. However, in the statement, which was focused on whether they would be able to accept the emotional support that AAI can provide, there were no such 100% responses but only slight agreement was given with this statement. Thus, there seems to be a gap between when a respondent expresses an opinion on a therapy that they do not participate in but is being applied on their close ones or when the respondent is an employee and objectively sees the impact of AAI on clients or when the respondent is actively receiving an AAI.

The perception by clients was mostly very good. Similar to the study by Pruskowski et al. [23], where patients from the burn unit were happy to participate in AAI and glad to continue the therapy, the clients in this study were also generally satisfied. For example, 94% of clients completely agreed with the statement that the presence of a dog dilutes the monotonous course of days in the facility, which can be considered a great result because this is also one of the reasons why AAI takes place in wards [46]. Specifically, 75% of clients also completely agreed that the presence of a dog has a positive effect on their mood, which is also another goal of AAI. This fact was also demonstrated in the study by Machová et al. [12], where patients also pointed to mood improvement after AAI. Similar results were obtained for the statement concerning the respondents’ enjoyment of AAI. Thus, it seems that AAI in the facilities where this study took place serves its purpose.

According to the results, 56% of clients completely trust the handler to control their dog, compared to 94% of employees. Another 44% of clients stated they slightly agree that the handler controls their dog. This finding seems to be related to the fact that clients are direct recipients of AAI, and their concerns are probably greater. However, employees have already seen dozens of AAIs, and it can be assumed that their opinion is based on previous experience. In the future, it would seem fitting to better inform clients, e.g., about the certification of dogs and handlers for AAI performance, or that most dogs and handlers usually need to be retested every year, and their potential concerns may be alleviated. Overall, 59% of patients do not fear the emergence of hygienic issues or damaging the bed, but 9% expressed slight concerns and 31% said they could not assess the issue. For this reason, it is necessary to pay attention to clients’ knowledge and explain to them that the dog included in AAI is always combed and, in case of being dirty from outdoor, is also washed and blow-dried. Therefore, these problems should not occur, although the period of molting always brings complications in this area. 

Of the patients, only 44% strongly agreed with the statement that the memories of their last AAI were positive, and another 50% rather agreed with the statement. This result is surprising and means that handlers could consider improving the course of AAI. This is also related to the possible differences among clients that may arise and which need to be taken into account. For example, when dealing with a man, it is better to actively seek him out and offer him this opportunity. Further, in older clients residing in a village, there is an opportunity for a broader description of AAI, because many of these respondents said that they cannot judge some statements. Additionally, clients with a high school diploma and postgraduate education receive AAI better than university students and so on. This offers opportunities to improve AAI itself and the approach to individual clients.

### 4.3. Limitations

The first limitation, but also an advantage of this study is the diversity of respondents. Some were directly involved in AAI, some were more passively involved in AAI, and for some, AAI is part of their work regime. However, the study aimed to evaluate the attitude of all directly and indirectly involved persons who are affected by AAI with a dog. We are also aware that the number of clients representing the main interest group is low. However, thanks to the evaluation of the point of view of all participants and taking into account their roles, we were just able to see their difference of opinion. Another limitation is a certain bias of some statements. For example, when asked whether respondents prefer a smaller breed, the statement does not include the possibility that they should prefer a large or medium breed. Some statements are suitable directly for active AAI participants, but this defect was made possible by the division of the group into individual roles. The authors also retrospectively evaluate the list of statements as too long, but the aim was to include most of the important statements, the evaluation of which is important for the practical performance of AAI. The study is, therefore, extensive and more difficult to navigate. Additionally, a larger number of respondents would certainly provide even stronger results. 

## 5. Conclusions 

Animal-assisted intervention is generally very well received. There may be some differences when considering factors such as respondents’ age, role in AAI, gender, size of settlement, or education. In general, AAI is better accepted by women, people who have completed postgraduate education or with a high school diploma, younger people from small towns or cities, and especially in the role of employees of facilities where AAI takes place. In some cases, clients reported lower evaluation of statements than, e.g., staff, and it appears that many of the results may be based on their lack of awareness of AAI’s course and rules. The results obtained could help to better understand the possible perception of AAI from different perspectives. In the future, it seems appropriate to focus in more detail on the perception of AAI by patients to improve the AAI methodology and awareness about it.

## Figures and Tables

**Table 1:** Table of dependent factors after rounding.

Question	Influencing Factors				
	Role	Sex	Education (Merged 0 + 1)	Residence	Age
1. I consider the presence of the therapy dog in healthcare and social care facilities as beneficial	-	*p* = 0.148	-	-	-
2. The therapy dog in healthcare and social care facilities brings a pleasure.	-	*P* = 0.763	-	-	-
3. The therapy dog can bring illnesses and parasites to healthcare and social care facilities.	-	*p* = 0.244	-	-	-
4. The therapy dog can cause allergies, what could complicate the functioning of the healthcare and social care facilities.	-	*p* = 0.299	*p* = 0.105	*p* = 0.846	*p* = 0.012 * V = 0.230
5. The presence of the therapy dog lives up monotonous healthcare and social care facilities stay.	*p* = 0.003 * V = 0.235	*p* < 0.001 * Phi = 0.246	*p* = 0.115	-	-
6. The presence of the therapy dog is not beneficial for the clients.	-	-	-	-	-
7. The presence of the therapy dog reduce the feeling of loneliness.	-	-	-	-	-
8. When I see the therapy dog, I am missing my own dog and I feel sorry that I cannot have my pet by my side.	-	*p* = 0.011 * V = 0.205	-	-	-
9. I would appreciate to have AAI settings more often.	-	*p* = 0.158	-	-	-
10. The presence of the therapy dog makes me feel nervous and anxious.	-	-	-	-	-
11. I believe that the owner of the therapy dog has the dog under the control during AAI.	*p* < 0.001 * V = 0.261	*p* = 0.004 * Phi = 0.199	*p* = 0.231	*p* = 0.163	*p* = 0.829
12. I am delighted when I think about the next AAI setting.			-	-	-
13. The activities with the therapy dog lift my spirit.	*p* = 0.033 * V = 0.179	*p* = 0.010 * Phi = 0.177	*p* = 0.376	*p* = 0.148	*p* = 0.096
14. I prefer the individual AAI rather than the group therapy.	-	*p* = 0.083	*p* = 0.255	*p* = 0.382	-
15. The presence of the therapy dog troubles the staff and stresses them out.	-	*p* = 0.024 * V = 0.187	*p* = 0.020 * V = 0.187	*p* = 0.502	-
16. The presence of the dog relaxes the clients in healthcare and social care facilities.	-	*p* = 0.977	*p* = 0.027 * V = 0.182	*p* = 0.547	-
17. I am looking forward to the next AAI setting.	*p* = 0.033 * V = 0.178	*p* = 0.015 * Phi = 0.167	*p* = 0.459	-	-
18. My memories of the last meeting with handler and therapy dog are positive.	*p* < 0.001 * V = 0.243	*p* = 0.021 * V = 0.191	*p* = 0.0795	*p* = 0.523	*p* = 0.339
19. The therapy dog causes fear of hygienic issues or worries about damaging the healthcare and social care facilities, bed or room.	*p* < 0.001 * V = 0.203	*p* < 0.001 * V = 0.413	*p* = 0.189	-	-
20. The therapy dog is suitable instrument to get in touch with the surrounding.	*p* < 0.001 * V = 0.242	*p* < 0.001 * V = 0.408	*p* = 0.002 * V = 0.223	*p* = 0.443	-
21. I consider the presence of the therapy dog in healthcare and social care facilities as inappropriate.	-	*p* < 0.001 * V = 0.331	-	-	-
22. One can observe that staff enjoys the presence of the therapy dog.	-	*p* < 0.001 * V = 0.335	*p* = 0.204	-	-
23. The therapy dog is suitable for the work with psychiatric patients.	-	*p* < 0.001 * V = 0.551	*p* < 0.001 * V = 0.254	*p* =0.038 * V = 0.154	-
24. It would bother me, if a volunteer visited me with the dog.	-	*p* = 0.004 * V = 0.227	*p* = 0.313	-	-
25. When I know, that the therapy dog will be present in the healthcare and social care facilities, I am actively looking for the dog’s presence.	*p* = 0.010 * V = 0.177	*p* < 0.001 * V = 0.489	*p* = 0.025 * V = 0.184	*p* = 0.707	*p* = 0.096
26. I prefer smaller dog breeds for the AAI.	-	*p* < 0.001 * V = 0.330	*p* = 0.085	*p* = 0.551	-
27. The AAI had positive impact on my psychological condition while my healthcare and social care facilities stays.	-	-	-	-	-
28. AAI helped me to reduce my stress level.	-	-	-	-	-
29. I am against animal-assisted interventions.	-	-	-	-	-
30. The presence of the therapy dog compensates me the absence of my pet.	-	-	-	-	-
31. Clients respect the presence of the therapy dog and are friendly to the dog.	*p* = 0.002 * V = 0.199	*p* < 0.001 * V = 0,457	*p* = 0.096	*p* = 0.001* V = 0.204	*p* = 0.432
32. I am worried that the work of the therapy dog is too demanding for the dog.	-	*p* < 0.001 * V = 0.384	*p* = 0.054	*p* = 0.506	-
33. Therapy dog is an ideal motivating element during therapy.	*p* = 0.00000 * V = 0.332	*p* < 0.001 * V = 0.441	*p* = 0.012 * V = 0.196	*p* < 0.001 * V = 0.251	*p* < 0.001 * V = 0.293
34. The presence of a therapy dog in the healthcare and social care facilities creates friendly and less formal atmosphere.	*p* < 0.001 * V = 0.347	*p* < 0.001 * V = 0.674	*p* < 0.001 * V = 0.248	*p* < 0.001 * V = 0.295	*p* < 0.001 * V = 0.261
35. I am more active during the presence of the therapy dog and the dog motivates me to exercise.	*p* < 0.001 * V = 0.351	*p* < 0.001 * V = 0.395	*p* = 0.035 * V = 0.168	-	-
36. The therapy dog could provide me some emotional support.	*p* < 0.001 * V = 0.502	*p* < 0.001 * V = 0.329	-	-	-
37. Petting and touching the therapy dog makes me calm down.	-	*p* < 0.001 * V = 0.366	-	-	-
38. I like to use the option of going for a walk with the therapy dog.	-	-	-	-	-
39. The presence of the therapy dog in the healthcare and social care facilities makes me doubt about the hygienic conditions.	-	-	-	-	-
40. If there is an option to join the program with the therapy dog, I take part in it.	-	-	-	-	-
41. I mind the presence of the therapy dog in the healthcare and social care facilities even if the therapy does not concern me.	-	*p* < 0.001 * V = 0.527	-	-	-
42. Even if the therapy program does not concern me, I agree with its implementation.	-	*p* = 0.004 * Phi = 0.198	-	-	-
43. During the contact with the therapy dog, I have better mood and this feeling lasts even after the therapy.	-	-	-	-	-
44. The dog breed influences the quality of the AAI.	-	-	-	-	-
45. I do not care about the presence of the therapy dog in the healthcare and social care facilities.	-	-	-	-	-

*Note.* * Statistically significant correlation was observed. A *p*-value < 0.05 is considered statistically significant. Symbol dash “-” was used, where no test was possible for different reasons, mostly for small sample size. In the case of a proven statistically significant dependence, the strength of this dependence was measured and assessed using Pearson’s contingency coefficient or Cramer’s contingency coefficient (for cases where one variable had more than 2 categories) and association coefficient and coefficient φ—Phi coefficient (for cases where each variable had exactly 2 categories).

**Table 2:** Age as a factor significantly influencing opinions and attitudes to AAT.

		Age Group (AG)/Row Percent					
Statement	Opinion	AG < 30; *N* = 44	AG 30–40; *N* = 31	AG 40–50; *N* = 56	AG 50–60; *N* = 41	AG 60–70; *N* = 21	AG > 70; *N* = 21
S4	0	29.55%	32.26%	25.00%	19.51%	28.57%	23.81%
	1	31.82%	9.68%	8.93%	9.76%	0.00%	9.52%
	2	38.64%	58.06%	66.07%	70.73%	71.43%	66.67%
S33	0	29.55%	16.13%	39.29%	29.27%	47.62%	42.86%
	2	29.55%	12.90%	14.29%	17.07%	42.86%	47.62%
	3	40.91%	70.97%	46.43%	53.66%	9.52%	9.52%
S34	0	18.18%	12.90%	32.14%	24.39%	38.10%	42.86%
	1	2.27%	9.68%	1.79%	12.20%	4.76%	4.76%
	2	22.73%	9.68%	12.50%	17.07%	38.10%	47.68%
	3	56.82%	67.74%	53.57%	46.34%	19.05%	4.76%

*Note:* abbreviation AG means age group; level of agreement with the statement (opinion) 0–1; 0—I cannot evaluate, 1—I do not agree, 2—I agree moderately, and 3—I completely agree.

**Table 3:** Residence as a factor significantly influencing opinions and attitudes to AAT.

		Residence (R)/Row Percent		
Statement	Opinion	R 1; *N* = 32	R 2; *N* = 162	R 3; *N* = 20
S23	0	40.63%	25.93%	50.00%
	2	18.75%	10.49%	5.00%
	3	40.63%	63.58%	45.00%
S31	0	34.38%	25.31%	20.00%
	2	15.63%	11.11%	45.00%
	3	50.00%	63.58%	35.00%
S33	0	40.63%	29.63%	50.00%
	2	50.00%	18.52%	25.00%
	3	9.38%	51.85%	25.00%
S34	0	43.75%	21.60%	40.00%
	1	6.25%	5.56%	5.00%
	2	46.88%	15.43%	25.00%
	3	3.31%	57.41%	30.00%

*Note:* abbreviation R means residence; R 1—villages with up to 500 inhabitants, R 2—city with 500–10,000 inhabitants, and R 3—city with more than 10,000 inhabitants; level of agreement with the statement (opinion) 0–1; 0—I cannot evaluate, 1—I do not agree, 2—I agree moderately, and 3—I completely agree.

**Table 4:** Education as a factor significantly influencing opinions and attitudes to AAT.

		Education(E)/Row Percent				
Statement	Opinion	E 0; *N* = 2	E 1; *N* = 39	E 2; *N* = 110	E 3; *N* = 35	E 4; *N* = 28
S15	0	50.00%	69.00%	57.00%	63.00%	36.00%
	1	50.00%	0.00%	4.00%	14.00%	11.00%
	2	0.00%	31.00%	39.00%	23.00%	54.00%
S16	0	0.00%	13.00%	6.00%	9.00%	4.00%
	2	0.00%	15.00%	35.00%	11.00%	39.00%
	3	100%	72.00%	58.00%	80.00%	57.00%
S20	0	0.00%	13.00%	23.00%	11.00%	4.00%
	2	0.00%	15.00%	27.00%	11.00%	7.00%
	3	100.00%	72.00%	27.00%	77.00%	89.00%
S23	0	50.00%	26.00%	27.00%	37.00%	14.00%
	2	0.00%	0.00%	27.00%	6.00%	0.00%
	3	50.00%	74.00%	27.00%	57.00%	86.00%
S25	0	0.00%	31.00%	27.00%	29.00%	32.00%
	2	50.00%	8.00%	27.00%	23.00%	4.00%
	3	50.00%	62.00%	27.00%	49.00%	64.00%
S33	0	50.00%	28.00%	27.00%	29.00%	25.00%
	2	50.00%	21.00%	27.00%	26.00%	4.00%
	3	0.00%	51.00%	27.00%	46.00%	71.00%
S34	0	50.00%	18.00%	27.00%	26.00%	7.00%
	1	0.00%	0.00%	27.00%	3.00%	0.00%
	2	50.00%	21.00%	27.00%	20.00%	4.00%
	3	0.00%	62.00%	27.00%	51.00%	89.00%
S35	0	50.00%	21.00%	27.00%	26.00%	7.00%
	1	0.00%	0.00%	27.00%	3.00%	4.00%
	2	50.00%	44.00%	27.00%	54.00%	68.00%
	3	0.00%	36.00%	27.00%	17.00%	21.00%

*Note:* abbreviation E means education; E 1—primary and secondary education without graduation, E 2—higher education, E 3—secondary education with graduation, and E 4—postgraduate education; level of agreement with the statement (opinion), 0–3; 0—I cannot evaluate, 1—I do not agree, 2—I agree moderately, and 3—I completely agree.

**Table 5:** Gender as a factor significantly influencing opinions and attitudes to AAT.

		Sex/Row Percent				Sex/Row Percent	
Statement	Opinion	Male; *N* = 98	Female; *N* = 116	Statement	Opinion	Male; *N* = 98	Female; *N* = 116
S10	2	9.18%	0.86%	S25	0	17.35%	35.34%
	3	66.33%	77.59%		2	43.88%	3.45%
S11	2	41.84%	23.28%		3	38.78%	61.21%
	3	58.16%	76.72%	S26	0	9.18%	2.59%
S13	0	23.47%	10.34%		2	39.80%	15.52%
	3	76.53%	89.66%		3	51.02%	81.90%
S15	0	65.31%	50.86%	S31	0	43.88%	11.21%
	1	2.04%	9.48%		2	21.43%	9.48%
	2	32.65%	39.66%		3	34.69%	79.31%
S17	2	21.43%	9.48%	S32	0	50.00%	33.62%
	3	78.57%	90.52%		1	15.31%	0.00%
S18	0	24.49%	29.31%		2	34.69%	66.38%
	2	32.65%	16.38%	S33	0	48.98%	19.83%
	3	42.86%	54.31%		2	31.63%	17.24%
S19	0	33.67%	7.76%		3	19.39%	62.93%
	1	48.98%	87.07%	S34	0	46.94%	9.48%
	2	17.35%	5.17%		1	12.24%	0.00%
S20	0	27.55%	6.90%		2	29.59%	13.79%
	2	29.59%	11.21%		3	11.22%	76.72%
	3	42.86%	81.90%	S35	0	36.73%	10.34%
S21	0	27.55%	6.90%		1	12.24%	2.59%
	1	64.29%	91.38%		2	35.71%	59.48%
	2	8.60%	1.72%		3	15.31%	27.59%
S22	0	61.22%	89.66%	S36	0	21.43%	1.72%
	1	30.61%	8.62%		2	60.20%	82.76%
	3	8.16%	1.72%		3	18.37%	15.52%
S23	0	44.90%	18.10%	S37	2	24.49%	0.86%
	2	24.49%	0.00%		3	75.51%	99.14%
	3	30.61%	81.90%	S41	0	32.65%	0.00%
S24	0	13.27%	1.72%		1	55.10%	98.28%
	1	67.35%	78.45%		2	12.24%	1.72%
	2	19.39%	19.83%	S42	0	11.22%	1.72%
					3	88.78%	98.28%

*Note:* level of agreement with the statement (opinion) 0–3; 0—I cannot evaluate, 1—I do not agree, 2—I agree moderately, and 3—I completely agree.

**Table 6:** Role of participants as a factor significantly influencing opinions and attitudes towards AAT.

			Role(R)/Row Percent		
Statement	Opinion		R 1;	R 2; *N* = 146	R 3; *N* = 36
			*N* = 32		
S5	2		6.00%	21.00%	0.00%
	3		94.00%	79.00%	100.00%
S11	2		44.00%	36.00%	6.00%
	3		56.00%	64.00%	94%
S13	0		25.00%	18.00%	3.00%
	3		75.00%	82.00%	97.00%
S17	2		25.00%	16.00%	3.00%
	3		75.00%	84.00%	97.00%
S18	0		6.00%	35.00%	14.00%
	2		50.00%	20.00%	17.00%
	3		44.00%	45.00%	69.00%
S19	0		31.00%	21.00%	3.00%
	1		59.00%	65.00%	97.00%
	2		9.00%	14.00%	0.00%
S20	0		25.00%	18.00%	0.00%
	2		22.00%	24.00%	0.00%
	3		53.00%	58.00%	100.0%
S25	0		16.00%	29.00%	28.00%
	2		34.00%	24.00%	3.00%
	3		50.00%	47.00%	69.00%
S31	0		25.00%	31.00%	8.00%
	2		22.00%	16.00%	3.00%
	3		53.00%	53.00%	89.00%
S33	0		41.00%	38.00%	8.00%
	2		47.00%	24.00%	3.00%
	3		13.00%	38.00%	89.00%
S34	0		37.50%	29.45%	5.56%
	1		6.25%	6.85%	0.00%
	2		46.88%	19.86%	2.78%
	3		9.38%	43.84%	91.67%
S35	0		28.00%	26.00%	3.00%
	1		9.00%	8.00%	0.00%
	2 3		63.00% 0.00%	34.00% 32.00%	16.00% 0.00%
S36	0		13.00%	12.00%	3.00%
	2		9.00%	82.00%	92.00%
	3		78.00%	6.00%	6.00%

*Note:* abbreviation R means role; Role 1—clients, Role 2—family member, and Role 3—staff; level of agreement with the statement (opinion) 0–1; 0—I cannot evaluate, 1—I do not agree, 2—I agree moderately, and 3—I completely agree.

## References

[1] Reed R., Ferrer L., Villegas N. (2012). Natural healers: A review of animal assisted therapy and activities as complementary treatment for chronic conditions. Rev. Lat. Am. Enfermagem..

[2] Cirulli F., Borgi M., Berry A., Francia N., Alleva E. (2011). Animal-assisted interventions as innovative tools for mental health. Ann. Ist. Super. Sanita..

[3] Balluerka N., Muela A., Amiano N., Caldentey M. (2014). Influence of Animal-Assisted Therapy (AAT) on the Attachment Representations of Youth in Residential Care. Child. Youth Serv. Rev..

[4] Dimitrijević I. (2009). Animal-Assisted Therapy—A New Trend In The Treatment of Children and Adults. Psychiatr. Danub..

[5] Muñoz Lasa S., Máximo Bocanegra N., Valero Alcaide R., Atín Arratibel M.A., Varela Donoso E., Ferriero G. (2015). Intervenciones asistidas por animales en neurorrehabilitación: Una revisión de la literatura más reciente. Neurología.

[6] Hediger K., Thommen S., Wagner C., Gaab J., Hund-Georgiadis M. (2019). Effects of animal-assisted therapy on social behaviour in patients with acquired brain injury: A randomised controlled trial. Sci. Rep..

[7] Souter M.A., Miller M.D. (2007). Do Animal-Assisted Activities Effectively Treat Depression? A Meta-Analysis. Anthrozoös.

[8] Ichitani T., Cunha M.C. (2016). Effects of animal-assisted activity on self-reported feelings of pain in hospitalized children and adolescents. Psicol. Refl. Crít..

[9] Schuck S.E.B., Johnson H.L., Abdullah M.M., Stehli A., Fine A.H., Lakes K.D. (2018). The Role of Animal Assisted Intervention on Improving Self-Esteem in Children with Attention Deficit/Hyperactivity Disorder. Front. Pediatr..

[10] Walsh F. (2009). Human-Animal Bonds I: The Relational Significance of Companion Animals. Fam. Process.

[11] Machová K., Součková M., Procházková R., Vaníčková Z., Mezian K. (2019). Canine-Assisted Therapy Improves Well-Being in Nurses. Int. J. Environ. Res. Public Health.

[12] Machová K., Procházková R., Eretová P., Svobodová I., Kotík I. (2019). Effect of Animal-Assisted Therapy on Patients in the Department of Long-Term Care: A Pilot Study. Int. J. Environ. Res. Public Health.

[13] Lefebvre S.L., Golab G.C., Castrodale L., Aureden K., Bialachowski A., Gumley N., Robinson J., Peregrine A., Benoit M., Card M.L. (2008). Guidelines for animal-assisted interventions in health care facilities. Am. J. Infect. Control.

[14] Jegathessan B. (2014). The IAHAIO Definitions for Animal Assisted Intervention and Guidelines for Wellness of Animals Involved. https://iahaio.org/wp/wp-content/uploads/2017/05/iahaio-white-paper-final-nov-24-2014.pdf.

[15] Cipriani J., Cooper M., DiGiovanni N.M., Litchkofski A., Nichols A.L., Ramsey A. (2013). Dog-Assisted Therapy for Residents of Long-Term Care Facilities: An Evidence-Based Review with Implications for Occupational Therapy. Phys. Occup. Ther. Geriatr..

[16] Lundqvist M., Carlsson P., Sjödahl R., Theodorsson E., Levin L.-Å. (2017). Patient benefit of dog-assisted interventions in health care: A systematic review. BMC Complement. Altern. Med..

[17] Moreira R.L., Gubert F.d.A., de Sabino L.M.M., Benevides J.L., Tomé M.A.B.G., Martins M.C., Brito M.d.A. (2016). Assisted therapy with dogs in pediatric oncology: Relatives’ and nurses’ perceptions. Rev. Bras. Enferm..

[18] Brisson S., Dekker A.H. (2017). Staff Attitudes Regarding the Impact of a Therapy Dog Program on Military Behavioral Health Patients. J. Spec. Oper. Med..

[19] Hinic K., Kowalski M.O., Holtzman K., Mobus K. (2019). The Effect of a Pet Therapy and Comparison Intervention on Anxiety in Hospitalized Children. J. Pediatr. Health Care.

[20] Gagnon J., Bouchard F., Landry M., Belles-Isles M., Fortier M., Fillion L. (2004). Implementing a hospital-based animal therapy program for children with cancer: A descriptive study. RCSIO/CONJ.

[21] Bouchard F., Landry M., Belles-Isles M., Gagnon J. (2004). A magical dream: A pilot project in animal-assisted therapy in pediatric oncology. RCSIO/CONJ.

[22] Nahm N., Lubin J., Lubin J., Bankwitz B.K., Castelaz M., Chen X., Shackson J.C., Aggarwal M.N., Totten V.Y. (2012). Therapy dogs in the emergency department. West. J. Emerg. Med..

[23] Pruskowski K.A., Gurney J.M., Cancio L.C. (2020). Impact of the implementation of a therapy dog program on burn center patients and staff. Burns.

[24] Barker S.B., Dawson K.S. (1998). The effects of animal-assisted therapy on anxiety ratings of hospitalized psychiatric patients. Psychiat. Serv..

[25] Kanamori M., Suzuki M., Yamamoto K., Kanda M., Matsui Y., Kozima E., Takeuchi S., Oshiro H. (2001). Evaluation of animal-assisted therapy for the elderly with senile dementia in a day care program. Geriatr. Gerontol. Int..

[26] Nagengast S.L., Baun M.M., Megel M., Leibowitz J.M. (1997). The effects of the presence of a companion animal on physiological arousal and behavioral distress in children during a physical examination. J. Pediatr. Nurs..

[27] Crowley-Robinson P., Blackshaw J.K. (1998). Nursing home staffs’ empathy for a missing therapy dog, their attitudes to animal-assisted therapy programs and suitable dog breeds. Anthrozoös.

[28] Gazzano A., Zilocchi M., Massoni E., Mariti C. (2013). Dogs’ features strongly affect people’s feelings and behavior toward them. J. Vet. Behav..

[29] Silva N.B., Osório F.L. (2018). Impact of an animal-assisted therapy programme on physiological and psychosocial variables of paediatric oncology patients. PLoS ONE.

[30] Jofré M. (2005). Visita terapéutica de mascotas en hospitales. Rev. Chilena. Infectol..

[31] Moody W.J., King R., O’ROURKE S. (2002). Attitudes of paediatric medical ward staff to a dog visitation programme. J. Clin. Nurs..

[32] Banks M.R., Banks W.A. (2005). The effects of group and individual animal-assisted therapy on loneliness in residents of long-term care facilities. Anthrozoös.

[33] Nimer J., Lundahl B. (2007). Animal-Assisted Therapy: A Meta-Analysis. Anthrozoös.

[34] Barker S.B., Pandurangi A.K., Best A.M. (2003). Effects of animal-assisted therapy on patients’ anxiety, fear, and depression before ECT. J. ECT.

[35] Wu A.S., Niedra R., Pendergast L., McCrindle B.W. (2002). Acceptability and impact of pet visitation on a pediatric cardiology inpatient unit. J. Pediatr. Nurs..

[36] Motooka M., Kennedy N.L., Koike H., Yokoyama T. (2006). Effect of dog-walking on autonomic nervous activity in senior citizens. Med. J. Australia.

[37] Schuelke S., Trask B., Wallace C., Baun M., Bergstrom N., McCabe B. (1991). Physiological effects of the use of a companion animal dog as a cue to relaxation in diagnosed hypertensives. Latham Letter.

[38] Arsenault P.M. (2004). Validating generational differences: A legitimate diversity and leadership issue. Leadership Org. Dev. J..

[39] Herzog H.A., Burghardt G.M. (1988). Attitudes toward Animals: Origins and Diversity. Anthrozoös.

[40] Gray P.B., Young S.M. (2011). Human–Pet Dynamics in Cross-Cultural Perspective. Anthrozoös.

[41] Gee N.R., Griffin J.A., McCardle P. (2017). Human–animal interaction research in school settings: Current knowledge and future directions. Aera Open.

[42] Weil J. (2015). Applying research methods to a gerontological population: Matching data collection to characteristics of older persons. Educ. Gerontol..

[43] Lindström Nilsson M., Funkquist E., Edner A., Engvall G. (2020). Children report positive experiences of animal-assisted therapy in paediatric hospital care. Acta Paediatr..

[44] Applebaum J.W., Peek C.W., Zsembik B.A. (2020). Examining U.S. pet ownership using the General Social Survey. J. Soc. Sci..

[45] Pinto A., De Santis M., Moretti C., Farina L., Ravarotto L. (2017). Medical practitioners’ attitudes towards animal assisted interventions. An Italian survey. Complement. Ther. Med..

[46] Phung A., Joyce C., Ambutas S., Browning M., Fogg L., Christopher B.-A., Flood S. (2017). Animal-assisted therapy for inpatient adults. Nursing 2020.

